# Whole-Genome Sequencing of Clinically Isolated Carbapenem-Resistant Enterobacterales Harboring *mcr* Genes in Thailand, 2016–2019

**DOI:** 10.3389/fmicb.2020.586368

**Published:** 2021-01-11

**Authors:** Wantana Paveenkittiporn, Watcharaporn Kamjumphol, Ratchadaporn Ungcharoen, Anusak Kerdsin

**Affiliations:** ^1^National Institute of Health, Department of Medical Sciences, Ministry of Public Health, Nonthaburi, Thailand; ^2^Faculty of Public Health, Kasetsart University, Nakhon, Thailand

**Keywords:** *mcr*, carbapenem-resistant Enterobacterales, Thailand, colistin, genome

## Abstract

Mobile colistin-resistant genes (*mcr*) have become an increasing public health concern. Since the first report of *mcr-1* in Thailand in 2016, perspective surveillance was conducted to explore the genomic characteristics of clinical carbapenem-resistant Enterobacterales (CRE) isolates harboring *mcr* in 2016–2019. Thirteen (0.28%) out of 4,516 CRE isolates were found to carry *mcr* genes, including 69.2% (9/13) of *E. coli* and 30.8% (4/13) of *K. pneumoniae* isolates. Individual *mcr-1.1* was detected in eight *E. coli* (61.5%) isolates, whereas the co-occurrence of *mcr-1.1* and *mcr-3.5* was seen in only one *E. coli* isolate (7.7%). No CRE were detected carrying *mcr-2, mcr-4*, or *mcr-5* through to *mcr-9*. Analysis of plasmid replicon types carrying *mcr* revealed that IncX4 was the most common (61.5%; 8/13), followed by IncI2 (15.4%; 2/13). The minimum inhibitory concentration values for colistin were in the range of 4–16 μg/ml for all CRE isolates harboring *mcr*, suggesting they have 100% colistin resistance. Clermont phylotyping of nine *mcr*-harboring carbapenem-resistant *E. coli* isolates demonstrated phylogroup C was predominant in ST410. In contrast, ST336 belonged to CC17, and the KL type 25 was predominant in carbapenem-resistant *K. pneumoniae* isolates. This report provides a comprehensive insight into the prevalence of *mcr*-carrying CRE from patients in Thailand. The information highlights the importance of strengthening official active surveillance efforts to detect, control, and prevent *mcr*-harboring CRE and the need for rational drug use in all sectors.

## Introduction

The global spread of carbapenem-resistant Enterobacterales (CRE) has become a leading public health concern. The lack of accessible treatment has resulted in the use of colistin, an outmoded antibiotic, as a last-resort therapeutic drug for human infections by gram-negative bacteria. The widespread use of colistin in humans and animals has led to the emergence of colistin resistance in gram-negative bacteria, and the rates of resistance are continuously increasing (Kempf et al., 2016; Elbediwi et al., 2019). In 2017, the World Health Organization acknowledged that CRE are a serious priority in a published list of globally important of 12 antimicrobial-resistant pathogens (World Health Organization, 2017). Of particular concern is the spread of *mcr* genes into CRE, which would create strains that are potentially pan-drug resistant (PDR).

A common mechanism of colistin resistance is thought to be associated with chromosomal mediation (Meletis and Skoura, 2018). The discovery of the first plasmid-mediated colistin resistance gene *mcr-1* in an *Escherichia coli* strain isolated from a pig in China led to an increasing number of reports on the identification of *mcr* in many bacterial species worldwide (Liu et al., 2016; Elbediwi et al., 2019). Thus far, 10 variants of *mcr* (*mcr-1* through to *mcr-10*) have been reported (Gharaibeh and Shatnawi, 2019; Wang et al., 2020). The *mcr* gene has been shown to encode a phosphoethanolamine transferase that alters lipid A in the lipopolysaccharide of the bacterial outer membrane by adding a phosphoethanolamine (Gharaibeh and Shatnawi, 2019). This reduces the attachment of colistin to the bacterial outer membrane and, therefore, prevents cell lysis.

The presence of *mcr* has been reported in different gram-negative bacteria isolated worldwide from animal and human sources (Elbediwi et al., 2019; Gharaibeh and Shatnawi, 2019). In Thailand, the first reported *mcr-1*-harboring *E. coli* were isolated from the stool of an asymptomatic person in 2012 (Olaitan et al., 2016). There has since been documentation of *mcr- 1-, mcr- 2-*, and *mcr-3*-carrying *E. coli* and *Klebsiella pneumoniae* strains isolated from patients (Paveenkittiporn et al., 2017; Srijan et al., 2018; Kamjumphol et al., 2019; Malchione et al., 2019). The current study investigated the genomic characterization of antimicrobial resistance genes and antimicrobial susceptibility of CRE isolated from patients in Thailand during a laboratory-based surveillance program in 2016–2019.

## Materials and Methods

### Identification of CRE and Detection of Antimicrobial Resistance Genes

**FIGURE 1:**
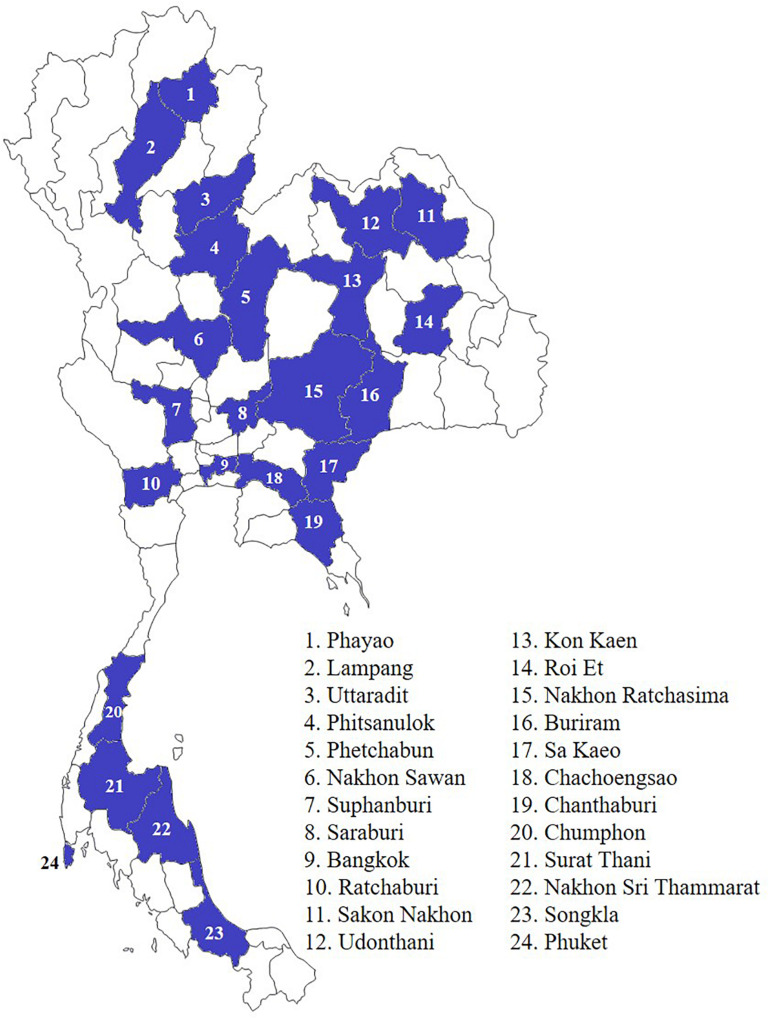
Location of hospital network in 24 provinces throughout Thailand.

In total, 6,996 multidrug-resistant (MDR) bacterial isolates were collected from individuals during 2016–2019 from a hospital network in 24 provinces throughout Thailand (Figure 1). There were 666, 2,763, 2,618, and 949 MDR isolates surveyed in 2016–2019, respectively. Conventional biochemical tests described elsewhere were used for the species identification of Enterobacterales (Abbott, 2011). The presence of carbapenemase (*bla*_IMP_, *bla*_KPC_, *bla*_NDM_, and *bla*_OXA–48–like_) and *mcr-1* genes was determined using multiplex PCR (Hatrongjit et al., 2018). The *mcr* genes (*mcr-1* to *mcr-9*) were detected using PCR, as described elsewhere (Rebelo et al., 2018; Wang et al., 2018; Yang et al., 2018; Yuan et al., 2019). All *mcr*-positive isolates were subjected to whole-genome sequencing.

### Antimicrobial Susceptibility Testing

Analysis of the minimal inhibitory concentration (MIC) of antimicrobials for *mcr*-harboring CRE isolates was performed using the Epsilometer test (*E* test) and interpreted according to the 2020 Clinical and Laboratory Standards Institute guidelines (Clinical and Laboratory Standards Institute, 2020), and *E. coli* ATCC 25922 was used as the control. The *E* test was based on ampicillin, amoxicillin-clavulanate, ampicillin-sulbactam, piperacillin-tazobactam, cefazolin, cefepime, cefotaxime, cefoxitin, ertapenem, meropenem, imipenem, gentamicin, amikacin, ciprofloxacin, levofloxacin, trimethoprim, fosfomycin, nitrofurantoin, chloramphenicol, tetracycline, aztreonam, and azithromycin. Additionally, a modified carbapenem inactivation method (mCIM) was applied to all CRE isolates according to 2020 CLSI M100-S30.

Broth microdilution was used to determine the MIC of colistin using colistin sulfate (Merck, Germany) at 1, 2, 4, 8, 16, and 32 μg/ml, respectively. According to 2020 CLSI M100-S30, an MIC of ≤2 μg/ml was interpreted as intermediate susceptibility, whereas an MIC of ≥4 μg/ml was considered to indicate resistance.

### Whole-Genome Sequencing and Analysis

DNA from 13 *mcr-*carrying CRE isolates was extracted from nutrient agar plate cultures using a DNeasy blood & tissue kit (Qiagen, Hilden, Germany) following the manufacturer’s recommendations, and the concentration was determined using the Qubit dsDNA BR assay kit (Invitrogen, Oregon, United States). Sequence libraries were prepared using a Qiagen QIAseq FX DNA library kit (Qiagen, Hilden, Germany) according to the manufacturer’s protocol. The prepared libraries were sequenced on the Illumina Miseq platform with Illumina MiSeq 2X 250 base paired-end chemistry (Illumina, CA, United States) according to the manufacturer’s instructions. The genomes for each strain were *de novo* assembled using CLC Genomics Workbench v12.0.2 (Qiagen, Aarhus, Denmark) with mostly default settings, except that the minimum contig size threshold was set to 500 bp.

Analysis of the whole-genome sequence data was performed as described elsewhere (Kerdsin et al., 2019). Briefly, the isolates were identified to species level using KmerFinder 3.1 (Larsen et al., 2014)^1^. Antimicrobial resistance genes were identified using ResFinder 4.1 (Zankari et al., 2012)^2^ and the Comprehensive Antibiotic Resistance Database (CARD) (Alcock et al., 2020)^3^. We investigated chromosome-mediated colistin resistance by analyzing the *mgrB*, *phoPQ*, *pmrAB, crrB*, and *rpoN* genes. The gene sequences were analyzed using local BLAST + and Clustal W, including *K. pneumoniae* MGH78578 (GenBank accession number NC_009648.1) and *E. coli* K12 sub-strain MG1655 (GenBank accession number U00096) genomes as colistin-susceptible references.

Plasmid replicons were analyzed using PlasmidFinder (Carattoli et al., 2014)^4^. Phylogrouping for *E. coli* and the KL type of *K. pneumoniae* were based on analysis using ClermonTyping (Beghain et al., 2018)^5^ and Kaptive (Wick et al., 2018)^6^. The virulence genes of *E. coli* and *K. pneumoniae* were analyzed using VirulenceFinder 2.0 (Joensen et al., 2014)^7^ and Institut Pasteur^8^, respectively.

For multilocus sequence typing (MLST) analysis of the sequence types (STs) of *E. coli* and *K. pneumoniae*, we used MLST 2.0 (Larsen et al., 2012)^9^. The genomic comparison of 13 *mcr*-harboring CRE isolates was conducted using a modular Single Genome Analysis to search for the genetically closest relatives in the database following the single nucleotide polymorphism (SNP) approach with BacWGSTdb (Feng et al., 2020)^10^. A phylogenetic tree was constructed using REALPHY and MEGA X via the neighbor-joining method with 500 bootstrap replicates by applying the Tamura three-parameter model (Bertels et al., 2014; Kumar et al., 2018). The tree was visualized and annotated using Interactive Tree of Life (ITOL) V4 (Letunic and Bork, 2016). *E. coli* K12 substrain MG1655 (accession no. U00096) and *K. pneumoniae* HS11286 (accession no. CP003200) were used as the reference sequences for SNP analysis. Details of the other genomes used for comparison with our isolates are shown in the Supplementary Materials.

### Statistical Analysis

The associations between *mcr* genes, *mcr*-harboring *E. coli* isolates, and non-*E. coli* isolates were analyzed by calculating the odds ratios (OR) and *p*-values using the STATA version 14 software package, with *p* < 0.05 considered to be statistically significant.

#### Accession Number

The assembled genomic sequences were deposited under the BioProject accession number PRJNA525849 with BioSample accessions: SAMN15497997-SAMN15498009. The accession numbers for each *mcr-1*-harboring CRE isolate are provided in Table 2.

## Results

### Genomic Analysis of CRE Isolates Harboring *mcr* Genes

Of the 6,996 MDR isolates, 4,516 were identified as CRE (64.5%). Of these, 4,235 (93.7%) isolates were classified as carbapenemase-producing Enterobacterales (CPE) and carried carbapenemase genes (*bla*_NDM_, *bla*_OXA–48–like_, *bla*_IMP_, or coexisting carbapenemase genes) according to the mCIM and PCR results. Of all the CPE isolates, 13 (0.3%) carried *mcr* genes (Table 1). The *mcr-*carrying rates among carbapenemase-producing *E. coli* and *K. pneumoniae* were 1.03 and 0.12%, respectively. Statistical analysis revealed a strong association between *mcr* and carbapenemase-producing *E. coli*, with the OR being 11.06 (95% CI, 3.07–49.23) and statistically significant (*p* < 0.0001) (Table 1).

**TABLE 1:** Distribution of *mcr* and carbapenemase genes in carbapenemase-producing Enterobacterales (CPE) during 2016–2019.

**Species**	**Total**	***bla*_NDM_**	***bla*_OXA –48 –like_**	***bla*_IMP_**	***bla*_NDM_ + *bla*_OXA –48 –like_**	***bla*_IMP_ + *bla*_NDM_**	***bla*_IMP_ + *bla*_OXA –48 –like_**	***bla*_NDM_*+ mcr***	***bla_OXA –48 –like_ + mcr***	***mcr-*carrying rate**	***p-value***	**Odd ratio (95% CI)**
*Escherichia coli*	868	695	54	1	108	1		8	1	1.03%	<0.0001	11.06 (3.07–49.23)
*Escherichia* sp.	2	1	1									
*Klebsiella pneumoniae*	3129	1206	1341	46	529	1	2	3	1	0.12%		
*Klebsiella aerogenes*	11	5	6									
*Klebsiella oxytoca*	3	3										
*Enterobacter cloacae*	189	108	24	37	12	3	5					
*Enterobacter* sp.	1	1										
*Citrobacter freundii*	10	9			1							
*Citrobacter* sp.	1	1										
*Morganella morganii*	1	1										
*Pantoea agglomerans*	4	3			1							
*Proteus mirabilis*	7	5		1	1							
*Proteus vulgaris*	2	2										
*Providencia* sp.	3	1	1		1							
*Salmonella enterica*	4	2		2								
	4235	2043	1427	87	653	5	7	11	2	0.31%		

**FIGURE 2:**
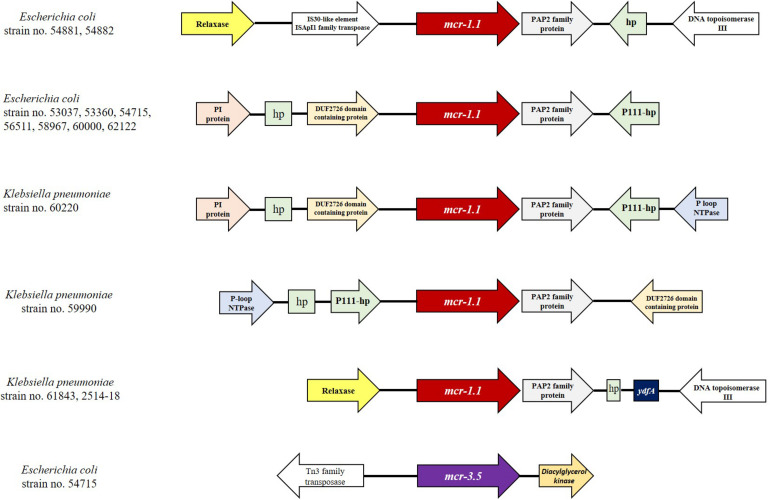
Comparison of the genetic organization of the *mcr* genes in 13 CRE isolates. The schematic shows the genes flanking the *mcr* genes in the isolates.

The proportions of *E. coli* and *K. pneumoniae* isolates showing *mcr* genes were 69.2% (9/13) and 30.8% (4/13), respectively. Individual *mcr-1.1* genes were detected in eight *E. coli* (61.5%) from 13 *mcr*-carrying CRE isolates, whereas the co-occurrence of *mcr-1.1* and *mcr-3.5* was found in only one *E. coli* isolate (7.7%) (Table 2). However, *mcr-2*, *mcr-4*, and *mcr-5* through to *mcr-9* were not detected. Analysis of the plasmid replicon types carrying *mcr* revealed that IncX4 was the most common (61.5%; 8/13), followed by IncI2 (15.4%; 2/13), as shown in Table 2. However, three isolates were unidentified. Six carbapenem-resistant *E. coli* isolates harboring *mcr* carried IncX4, whereas two isolates contained IncI2. Of the four carbapenem-resistant *K. pneumoniae* isolates harboring *mcr*, two carried IncX4 and the other two had unknown plasmid replicon types. The genetic organization of the *mcr* genes in these 13 isolates is outlined in Figure 2. A common gene found downstream of *mcr-1.1* in all isolates encoded the PAP2 family protein, whereas the upstream genes varied. However, a DUF2726-domain-containing protein-encoding gene was commonly found in 8 of the 13 isolates (seven *E. coli* and one *K. pneumoniae*). Furthermore, the upstream and downstream genetic organization of *mcr-3.5* was quite different from that of *mcr-1.1*.

**TABLE 2:** Distribution of sequence types and antimicrobial resistant genes in carbapenem-resistant *E. coli* and *K. pneumoniae* carrying *mcr* genes.

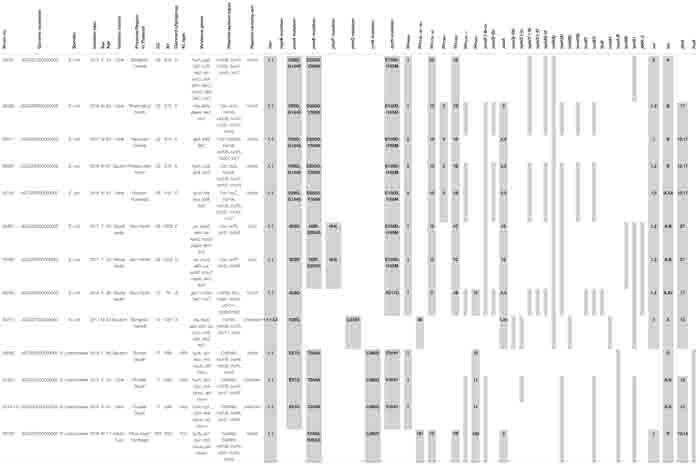

As shown in Table 2, chromosomal-mediated colistin resistance gene mutations, including those in *mgrB*, *pmrAB*, *phoPQ, crrB*, and *rpoN*, were analyzed. We detected substitutions in *pmrAB* and *rpoN* in almost *mcr*-harboring CRE isolates, whereas *phoPQ* substitutions were found in three isolates, and no mutations were detected in *mgrB.* Substitutions were commonly found in the *pmrA* genes of 12 out of 13 *mcr-*harboring isolates, while *pmrB* and *rpoN* substitutions were detected in 11 isolates. The isolates 54881 and 54882 contained more mutations in the chromosomal-mediated colistin resistance genes than other isolates. Substitution at S29G and E57G in *pmrA* was predominant in *E. coli* and *K. pneumoniae*, respectively. The *pmrB* substitution at D283G was commonly found in *E. coli*, whilst all four *mcr-1*-harboring *K. pneumoniae* contained a T246A substitution. Only one substitution in *phoP* (I44L) and one in *phoQ* (L343V) were detected in *mcr-1*-carrying *E. coli*. Substitution at E150D and I165M in *rpoN* was predominant in *E. coli* isolates, in contrast to those at F304Y which was commonly found in *K. pneumoniae* isolates. In addition, one *crrB* substitution (L296Q) was found in all *K. pneumoniae* isolates. Insertion or deletion in those described genes was not detected in all *mcr-1*-harboring CRE isolates.

As shown in Table 2, of the 13 isolates, five and three *mcr*-harboring *E. coli* isolates carried *bla*_NDM–1_ and *bla*_NDM–5_, respectively. Only one *E. coli* isolate contained *bla*_OXA–48_. Three and one *mcr-1.1*-harboring *K. pneumoniae* isolates carried *bla*_NDM–1_ and *bla*_OXA–181_, respectively. Among the β-lactamase genes, *bla*_CTX–M_, *bla*_SHV_, or *bla*_TEM_ were detected in almost all isolates (92.3%, 12/13), and only one *E. coli* isolate had no β-lactamase genes. The predominant *bla*_CTX–M_ was *bla*_CTX–M–15_, which was detected in 53.8% (7/13) of all isolates. The gene *bla*_SHV_ was found in all *K. pneumoniae* and one isolate of *E. coli* (Table 2). Among the ampC β-lactamase genes, *bla*_CMY–2_ only was detected in four *E. coli* isolates (28.6%). The other antimicrobial resistance genes in the *mcr*-carrying CRE isolates, including those for fluoroquinolones, aminoglycosides, rifampicin, macrolides, chloramphenicol, sulfonamide, tetracycline, fosfomycin, and trimethoprim, are shown in Table 2.

### Antimicrobial Susceptibility of CRE Isolates Harboring *mcr* Genes

As shown in Table 3, the colistin MIC values for the *mcr*-harboring isolates were in the range of 4–16 μg/ml. According to the 2020 CLSI M100-S30 guidelines, a microbe with a colistin MIC value of ≥4 μg/ml is resistant, whereas MIC ≤ 2 μg/ml indicates intermediate resistance. Thus, the results indicate that 100% of the *mcr*-carrying CRE isolated from patients were resistant to colistin. The highest MIC value for colistin (16 μg/ml) was found in two carbapenem-resistant *K. pneumoniae* isolates. Most of the *mcr*-harboring *E. coli* had colistin MIC values of 4 μg/ml (8/9; 88.8%).

**TABLE 3:** Antimicrobial susceptibility of *mcr*-harboring carbapenem-resistant *E. coli* and *K. pneumoniae*.

**No.**		**1**	**2**	**3**	**4**	**5**	**6**	**7**	**8**	**9**	**10**	**11**	**12**	**13**
Isolate No.		53360	54881	54882	56511	58967	62122	60000	54715	53037	59990	60220	61843	2514-18
Organism		*E. coli*	*E. coli*	*E. coli*	*E. coli*	*E. coli*	*E. coli*	*E. coli*	*E. coli*	*E. coli*	*K. pneumoniae*	*K. pneumoniae*	*K. pneumoniae*	*K. pneumoniae*
Classification		XDR	XDR	MDR	MDR	MDR	XDR	XDR	MDR	MDR	MDR	XDR	MDR	MDR
Penicillin	AMP (μg/ml)	>256 (R)	>256 (R)	>256 (R)	>256 (R)	>256 (R)	>256 (R)	>256 (R)	>256 (R)	>256 (R)	>256 (R)	>256 (R)	>256 (R)	>256 (R)
β-lactam combination	AMC (μg/ml)	>256 (R)	>256 (R)	>256 (R)	>256 (R)	>256 (R)	>256 (R)	>256 (R)	>256 (R)	>256 (R)	>256 (R)	>256 (R)	>256 (R)	>256 (R)
	SAM (μg/ml)	>256 (R)	>256 (R)	>256 (R)	>256 (R)	>256 (R)	>256 (R)	> 256 (R)	> 256 (R)	> 256 (R)	> 256 (R)	> 256 (R)	> 256 (R)	> 256 (R)
	TZP (μg/ml)	> 256 (R)	>256 (R)	> 256 (R)	>256 (R)	> 256 (R)	>256 (R)	> 256 (R)	64 (I)	> 256 (R)	>256 (R)	> 256 (R)	>256 (R)	> 256 (R)
13rd generation Cephalosporins	KZ (μg/ml)	>256 (R)	>256 (R)	>256 (R)	>256 (R)	>256 (R)	>256 (R)	>256 (R)	32 (R)	>256 (R)	>256 (R)	>256 (R)	>256 (R)	>256 (R)
	FEP (μg/ml)	>256 (R)	>256 (R)	>256 (R)	>256 (R)	>256 (R)	>256 (R)	>256 (R)	0125 (S)	>256 (R)	>256 (R)	>256 (R)	96 (R)	96 (R)
	CTX (μg/ml)	>32 (R)	>32 (R)	>32 (R)	>32 (R)	>32 (R)	>32 (R)	>32 (R)	0.25 (S)	>32 (R)	>32 (R)	>32 (R)	>32 (R)	>32 (R)
	FOX (μg/ml)	>256 (R)	>256 (R)	>256 (R)	>256 (R)	>256 (R)	>256 (R)	>256 (R)	8 (S)	>256 (R)	>256 (R)	>256 (R)	>256 (R)	>256 (R)
Carbapenems	ERT (μg/ml)	>32 (R)	>32 (R)	>32 (R)	>32 (R)	>32 (R)	>32 (R)	>32 (R)	0.75 (I)	32 (R)	>32 (R)	>4(R)	>32 (R)	>32 (R)
	MER (μg/ml)	>32 (R)	>32 (R)	>32 (R)	12(R)	12 (R)	8 (R)	>32 (R)	0.5 (S)	8 (R)	>32 (R)	>8(R)	>32 (R)	>32 (R)
	IMP (μg/ml)	>32 (R)	>32 (R)	>32 (R)	1.5 (I)	8 (R)	8 (R)	>32 (R)	0.25 (S)	4 (R)	32 (R)	>8 (R)	>32 (R)	>32 (R)
Aminoglycoside	CN (μg/ml)	64 (R)	32 (R)	0.38 (S)	0.75 (S)	32 (R)	16 (R)	>256 (R)	24 (R)	0.75 (S)	0.5 (S)	0.75 (S)	0.75 (S)	0.75 (S)
	AK (μg/ml)	32 (I)	4 (S)	4 (S)	8 (S)	6 (S)	3 (S)	3 (S)	2 (S)	3 (S)	2 (S)	32 (I)	4 (S)	6 (S)
Quinolone	CIP (μg/ml)	>32 (R)	>32 (R)	3 (R)	>32 (R)	>32 (R)	>32 (R)	>32 (R)	>32 (R)	>32 (R)	0.094 (S)	>32 (R)	1.5 (R)	1 (R)
	LEV (μg/ml)	>32 (R)	>32 (R)	0.75 (S)	>32 (R)	>32 (R)	>32 (R)	>32 (R)	>32 (R)	>32 (R)	0.125 (S)	>32 (R)	0.5 (S)	0.25 (S)
Folate	SXT (μg/ml)	>32 (R)	>32 (R)	>32 (R)	>32 (R)	>32 (R)	>32 (R)	>32 (R)	>32 (R)	0.38 (S)	0.38 (S)	>32 (R)	4 (R)	>32 (R)
Fosfomycin	FOT (μg/ml)	0.75 (S)	6 (S)	0.75 (S)	0.75 (S)	1 (S)	1.5 (S)	0.75 (S)	0.5 (S)	0.38 (S)	24 (S)	64 (S)	96 (S)	>256 (R)
Nitrofuran	N (μg/ml)	32 (S)	16 (S)	16 (S)	24 (S)	48 (S)	64 (I)	64 (I)	32 (S)	16 (S)	128 (R)	>512 (R)	96 (R)	>256 (R)
Phenicol	C (μg/ml)	24 (I)	>256 (R)	16 (I)	12 (S)	24 (I)	64 (R)	>256 (R)	128 (R)	6 (S)	32 (R)	96 (R)	8 (S)	3 (S)
Tetracycline	TE (μg/ml)	>256 (R)	>256 (R)	>256 (R)	>256 (R)	>256 (R)	>256 (R)	96 (R)	48 (R)	64 (R)	>256 (R)	>256 (R)	>256 (R)	>256 (R)
Monobactam	ATM (μg/ml)	>256 (R)	>256 (R)	>256 (R)	>256 (R)	>256 (R)	>256 (R)	>256 (R)	0.047 (S)	48 (R)	96 (R)	>256 (R)	0.064 (S)	0.125 (S)
Macrolide	AZM (μg/ml)	32 (R)	>256 (R)	>256 (R)	32 (R)	>256 (R)	>256 (R)	>256 (R)	32 (R)	>256 (R)	>256 (R)	>256 (R)	>256 (R)	32 (R)
Colistin	COL (μg/ml)	4 (R)	4 (R)	4 (R)	4 (R)	4 (R)	4 (R)	8 (R)	4 (R)	8 (R)	8 (R)	8 (R)	16 (R)	16 (R)

*AMP, ampicillin; AMC, amoxicillin/clavulanic acid; SAM, ampicillin-sulbactam; TZP, piperacillin-tazobactam; KZ, Cefazolin; FEP, cefepime; CTX, cefotaxime; FOX, Cefoxitin; ERT, ertapenem; MER, meropenem; IMP, imipenem; CN, gentamicin; AK, amikacin; CIP, ciprofloxacin; LEV, levofloxacin; SXT, trimethoprim; FOT, Fosfomycin; N, nitrofurantoin; C, chloramphenicol; TE, tetracycline; ATM, aztreonam; AZM, azithromycin; COL colistin.*

More than 50% of the *mcr*-harboring CRE isolates were susceptible to amikacin (11/13), fosfomycin (12/13), and nitrofurantoin (7/13). Of the 13 CRE-harboring *mcr* isolates, four *E. coli* isolates and one *K. pneumoniae* isolate were extensively drug-resistant (XDR; 38.5%). Details of the antimicrobial resistance profiles of the 13 isolates are provided in Table 3. Moreover, 12 of the 13 *mcr*-harboring CRE isolates were resistant to ciprofloxacin. As shown in Table 2, ciprofloxacin resistance may result from the presence of quinolone resistance genes: *oqxA, oqxB, qnrS1, qnrB6*, and *aac(6′)-Ib-cr*.

### Molecular Typing of *mcr*-Harboring CRE Isolates

As summarized in Table 2, Clermont phylotyping of nine *mcr*-harboring carbapenem-resistant *E. coli* isolates demonstrated that phylogroup C (5/9; 55.6%) was predominant, followed by phylogroups A (2/9; 22.2%) and D (2/9; 22.2%). The Clermont phylogroup was concordant with clonal complexes (CC). Phylogroup C was concordant with CC23, which contained only ST410. Phylogroup D was concordant with CC38, which consisted of ST3052, whereas phylogroup A was concordant with CC10, which contained either ST10 or ST1287. Four carbapenem-resistant *K. pneumoniae* isolates harboring *mcr* were predominantly ST336 (3/4; 75%) and one was ST340 (1/4; 25%). ST336 belonged to CC17 and KL type 25, whereas ST340 belonged to CC258 and KL type 15.

**FIGURE 3:**
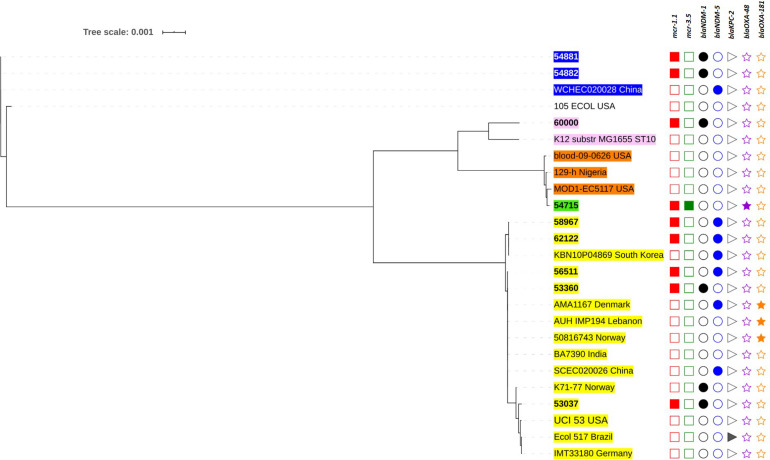
Phylogenetic tree based on single nucleotide polymorphisms (SNP) in *E. coli* using the neighbor-joining method. The tree was visualized and annotated using Interactive Tree of Life (iToL). The tree is annotated based on sequence types (STs; color highlight), *mcr* (square), *bla*_NDM_ (circle), *bla*_KPC_ (right triangle), and *bla*_OXA–48–like_ (star). Presentation of the genes is shown by filled symbols. STs 410, 1287, 617, 10, and 3052 are highlighted in yellow, green, brown, pink, and blue, respectively. The isolates used in this study are shown in bold.

**FIGURE 4:**
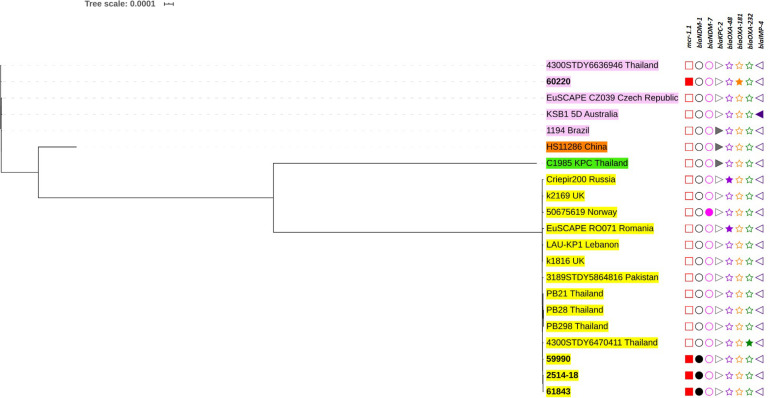
Phylogenetic tree based on single nucleotide polymorphisms (SNP) in *K. pneumoniae* using the neighbor-joining method. The tree was visualized and annotated using Interactive Tree of Life (iToL). The tree is annotated based on sequence types (STs; color highlight), *mcr* (square), *bla*_NDM_ (circle), *bla*_KPC_ (right triangle), *bla*_OXA–48–like_ (star), and *bla*_IMP_ (left triangle). Presentation of the genes is revealed by filled symbols. STs 336, 340, 11, and 4008 are highlighted in yellow, pink, and brown, and green, respectively. The isolates used in this study are shown in bold.

The genetic relationships based on the SNPs of these *mcr*-harboring isolates are demonstrated in Figures 3, 4. Five *E. coli* ST410 isolates were widely distributed in several sub-clusters of the ST410 cluster. Strain no. 53360 was closely related to the strain AMA1167 from Denmark, whereas strain no. 56511 clustered with strains from Norway, India, Lebanon, China, and Denmark (Figure 3). Strains no. 58967 and no. 62122 were related to strain KBN10P04869 from South Korea. Strain no. 53037 clustered with strains from Norway, the United States, Brazil, and Germany. Two ST3052 isolates (nos. 54881 and 54882) were closely related to the strain WCHEC020028 from China; they had similar characteristics and were from different individuals in the same hospital ward, indicating that they were likely to have originated from the same source. Strain no. 54715 (ST1287) was related to a strain from the United States (ST617), and these were clustered together with strain no. 6000 (ST10) (Figure 3).

Among the *K. pneumoniae* isolates, the ST336 isolates were closely related and clustered with other ST336 strains isolated in Thailand (Figure 4). Interestingly, the Thai-ST336 isolates were in a different cluster from that containing the ST336 isolates from other countries. Similarly, isolate no. 60220 (ST340) in this study was closely related to strain 4300STDY6636946 circulating in Thailand (Figure 4).

## Discussion

The discovery that plasmid-mediated colistin resistance is encoded by *mcr* genes and the high prevalence of human isolates harboring these genes are of global concern. A recent report revealed the overall average prevalence of *mcr* genes to be 4.7% (0.1–9.3%) in 47 countries across six continents (Elbediwi et al., 2019), and as many as 10 *mcr* genes (*mcr-1* through to *mcr-10)* have been reported (Gharaibeh and Shatnawi, 2019; Wang et al., 2020). A study of the global prevalence of *mcr* genes revealed that *mcr-1* (4917/5191; 94.7%) is a common gene and has a wider distribution compared with *mcr*-2 through to *mcr-8* (Elbediwi et al., 2019). Human infections with both CRE and non-CRE isolates carrying *mcr-1* have been widely reported (Liu et al., 2016; Mediavilla et al., 2016; Paveenkittiporn et al., 2017; Quan et al., 2017; Mendes et al., 2018; Srijan et al., 2018; Zhong et al., 2018; Elbediwi et al., 2019).

The coexistence of *mcr* and carbapenemase genes, such as *bla*_NDM_, *bla*_OXA–48–like_, and *bla*_IMP_, in CRE isolates has been described in countries worldwide (Mediavilla et al., 2016; Arabaci et al., 2019; Huang et al., 2020; Kananizadeh et al., 2020). The current study found the predominant *mcr* gene to be *mcr-1*, which more frequently coexists with *bla*_NDM_ than *bla*_OXA48–like_, highlighting the potential dissemination of *mcr-1* and *bla*_NDM_ among CRE isolates in Thailand. This concurs with a study in China (Huang et al., 2020), where *mcr-1* and *bla*_NDM–5_ were predominant (78.6%, 11/14). In this study, *mcr-1* and *bla*_NDM–1_ were the most prevalent resistance genes (61.5%, 8/13). A previous study showed that *mcr-3* had a wide distribution in water, animals, food, and human isolates (Elbediwi et al., 2019). We found a 7.7% (1/13) prevalence for *mcr-3.5*, which co-occurred with *mcr-1* and *bla*_OXA–48_ in *E. coli.* The phenomenon of double *mcr* genes has been reported in isolates from humans, with *K. pneumoniae* harboring *mcr-3* and *mcr-8* being isolated from the stool of a healthy individual in Laos (Hadjadj et al., 2019).

Our study revealed that the most common type of plasmid replicon carrying *mcr* was IncX4. Previous reports have shown IncX4, IncI2, and IncHI2 to be the major plasmid types driving the global dissemination of *mcr-1* (Wu et al., 2018). A study in Thailand revealed two predominant plasmid types (IncX4 and IncI2) carrying *mcr-1* in CRE (Shanmugakani et al., 2019). This suggests that IncX4 bearing *mcr-1* mediates the transmission of CRE and may promote its circulation throughout Thailand. IncX4 and IncI2 acting as vehicles for *mcr-1* propagation enhance host fitness and provide a competitive advantage over strains with other plasmid replicon types, resulting in greater plasmid stability (Wu et al., 2018).

On the basis of the Clermont phylotyping scheme, *E. coli* species can be divided into eight main phylogroups, termed A, B1, B2, C, D, E, F, and G (Clermont et al., 2019). The nine carbapenem-resistant *E. coli* isolates carrying *mcr* (55.6%) in this study belonged to phylogroup C, whereas the rest belonged to phylogroups A and D (22.2% each). The *E. coli* strains responsible for extra-intestinal infection were more likely to be members of phylogroups B2 or D, which show greater pathogenesis than A, B1, or C (Clermont et al., 2013). Strains belonging to phylogroups A, B1, and C are commonly commensal, suggesting that more than half of the *E. coli* harboring *mcr* isolated from patients in this study were commensal strains.

Our study revealed seven carbapenem-resistant STs that carry *mcr*, of which *E. coli* ST410 (35.7%) and *K. pneumoniae* ST336 (21.4%) isolates were predominant. Elbediwi et al. (2019) reported that *E. coli* ST101 carrying *mcr-1* have been found in environmental samples, animals, and humans. However, ST10 was the most globally common ST of *E. coli* carrying *mcr-1* (Elbediwi et al., 2019). In Asia, ST116 was found to be the predominant ST that carries *mcr*-1 isolated from humans, followed by ST117, ST10, ST38, ST101, and ST156 (Elbediwi et al., 2019). *E. coli* ST410 is internationally considered a new high-risk clone that can cause several types of infection; it is highly resistant and has a global distribution (Roer et al., 2018). This ST has been described in Southeast Asia following multiple introductions through several independent events and differs from clones detected in Europe and North America (Nadimpalli et al., 2019). SNP phylogenetic analysis in this study revealed that the ST410 isolates were diverse or not closely related to other strains. Instead, they were shown to be related to strains from countries other than Thailand. This adds support to the assumption that there have been multiple dissemination events into this area.

*Klebsiella pneumoniae* ST336 (CC17) is considered an international clone (Rodrigues et al., 2014; Novović et al., 2017; Palmieri et al., 2020) and has been frequently associated with the worldwide spread of *bla*_CTX–M–15_ and *bla*_OXA–48–like_ (Rodrigues et al., 2014; Novović et al., 2017; Palmieri et al., 2020). Interestingly, all ST336 isolates in the current study carried *bla*_NDM–1_, but not any *bla*_CTX–M_ genes, suggesting that they may be from different lineages. To the best of our knowledge, the carbapenem-resistant ST336 isolates in this study are the first to be described as having *mcr-1*. Previous studies revealed that colistin-resistant ST336 resulted from an *mgrB* mutation, and no *mcr* genes have been detected in this ST (Novović et al., 2017; Palmieri et al., 2020). SNP phylogenetic analysis allocated the ST336 isolates to the same cluster as other Thai-ST336 isolates, and this cluster was independent from another ST336 cluster consisting of isolates from other countries. This suggests that Thai-ST336 isolates circulate throughout our country by clonal expansion.

The colistin MIC values (4–16 μg/ml) for our isolates indicated they have 100% resistance. Combinations of *mcr* and chromosome-mediated colistin resistant genes (*pmrAB*, *phoPQ, crrB*, or *recN*) contributed to the colistin resistance of our isolates. It is interesting that substitutions in *pmrAB* and *crrB* in *mcr-1*-carrying *K. pneumoniae* are quite different from previous reports (Olaitan et al., 2014a,b; Wright et al., 2015; Cheng et al., 2016). In addition, substitution of *phoPQ* was not found in our *K. pneumoniae* isolates comparing to those studies (Olaitan et al., 2014a,b; Wright et al., 2015; Cheng et al., 2016). On the other hand, substitutions of *pmrA* (G144S), *pmrB* (H2R, D283G, Y358N), and *phoP* (I44L) in our *mcr-1*-harboring *E. coli* are similar to a study previously described (Choi et al., 2020). In case of *rpoN*, inactivation of this gene resulting in polymyxin resistance has been observed in *Salmonella enterica* (Barchiesi et al., 2009). However, polymyxin resistance via *rpoN* inactivation or substitution is not yet reported in either *E. coli* or *K. pneumoniae*. Although our study detected substitution of *rpoN*, but its role on polymyxin resistance remain to be investigated.

Several studies have shown *mcr-1*-carrying CRE isolated from humans to have high frequencies of colistin resistance, such as 71.4% in China (Huang et al., 2020), 100% in the United States (Mediavilla et al., 2016), and 100% in Turkey (Arabaci et al., 2019). A previous study in Thailand reported colistin resistance rates of 75.0 and 79.1% in carbapenem-resistant *E. coli* and *K. pneumoniae* isolates, respectively (Eiamphungporn et al., 2018). The 13 carbapenem-resistant *mcr-1*-harboring CRE isolates described here showed a high susceptibility (>50%) to the antibiotics amikacin, fosfomycin, and nitrofurantoin. In contrast, the human *mcr-1*-harboring CRE isolate from China was reported to be highly susceptible only to tigecycline, amikacin, and aztreonam (Huang et al., 2020), whereas the isolate from the United States was susceptible to more antibiotics, including amikacin, aztreonam, gentamicin, nitrofurantoin, tigecycline, and trimethoprim-sulfamethoxazole (Mediavilla et al., 2016).

Polymyxins, including colistin, were reintroduced into human medical practice by the WHO in 2012 (World Health Organization, 2012). In China, this antibiotic was approved for clinical use in the treatment of bacterial infections in 2017. Since then, the relative prevalence of CRE carrying *mcr* genes increased from 0.41 to 1.38% (Huang et al., 2020). A study in Singapore revealed that prior exposure to polymyxin (adjusted OR, 21.31; 95% CI, 3.04–150.96) and carbapenem (OR 3.74; CI 1.13–12.44) were independent risk factors for polymyxin-resistant CRE among hospitalized patients (Teo et al., 2019). A study in Thailand demonstrated that chronic kidney diseases (OR 3.95; CI 1.26–12.32) and exposure to antimicrobials for less than 3 months (OR 2.29; CI 0.29–18.21) were risk factors associated with infections by *mcr-1*-carrying Enterobacteriaceae (Shanmugakani et al., 2019). In patients infected with polymyxin-resistant CRE, the 30-day all-cause in-hospital mortality was 50.0% compared with a 38.1% mortality in patients with polymyxin-susceptible CRE (Teo et al., 2019). Therefore, minimizing the use of polymyxin and carbapenem is strongly recommended.

The findings of the current study provide comprehensive insights into the prevalence of *mcr*-carrying CRE in patients in Thailand. In general, *mcr-1* was present in *E. coli* and *K. pneumoniae* isolates. The co-occurrence of two *mcr* genes was also demonstrated in CRE isolated from patients. To slow the emergence of XDR or PDR strains, priority should be given to strengthening official surveillance, active control, and prevention efforts as well as minimizing the dissemination of *mcr* genes among CRE isolates in humans.

## Data Availability Statement

The datasets generated for this study can be found in the Raw sequencing data were deposited in the Sequence Read Archive (SRA) of NCBI under the BioProject ID PRJNA380676.

## Ethics Statement

The Human Research Ethics Committee of Department of Medical Sciences, Ministry of Public Health, reviewed this study and judged that the protocol constituted routine public health activities and therefore did not involve human subject research. Written informed consent for participation was not required for this study in accordance with the national legislation and the institutional requirements.

## Author Contributions

WP and AK conceived and designed the study, performed the data analysis, drafted the manuscript, and performed critical revisions of the manuscript for intellectual content. WK performed the laboratory experiments and analyzed the data. RU performed the statistical analysis and critical revision of the manuscript for intellectual content. All the authors read, edited, and approved the final manuscript.

## Conflict of Interest

The authors declare that the research was conducted in the absence of any commercial or financial relationships that could be construed as a potential conflict of interest.

## Footnotes

1 https://cge.cbs.dtu.dk/services/KmerFinder/

2 https://cge.cbs.dtu.dk/services/ResFinder/

3 https://card.mcmaster.ca/

4 https://cge.cbs.dtu.dk/services/PlasmidFinder/

5 http://clermontyping.iame-research.center/

6 http://kaptive.holtlab.net

7 https://cge.cbs.dtu.dk/services/VirulenceFinder/

8 https://bigsdb.pasteur.fr/klebsiella/klebsiella.html

9 https://cge.cbs.dtu.dk/services/MLST/

10 http://bacdb.org/BacWGSTdb/
