# Supplementary material for: Whole-Genome Sequencing of Clinically Isolated Carbapenem-Resistant Enterobacterales Harboring mcr Genes in Thailand, 2016–2019
Source: Front Microbiol. 2021 Jan 11;11:586368. doi: 10.3389/fmicb.2020.586368 (PMC7829498; doi:10.3389/fmicb.2020.586368)
Supplement: Supplementary file 1 [file Table_1.DOCX]

**Table S1.** Genome sequences of *Escherichia coli* and *Klebsiella pneumoniae* used in this study

| **Species** | **Strain no.** | **Accession no.** | **ST** | **Carbapenemase genes** | **Source of isolate** | **Country** |
| --- | --- | --- | --- | --- | --- | --- |
| *E. coli* | K71-77 | LNGY00000000 | 410 | *blaNDM-1* | Human: blood | Norway |
|  | BA7390 | PVPM00000000 | 410 | *-* | Human: blood | India |
|  | SCEC020026 | NGVF01000000 | 410 | *blaNDM-5* | Human: unknown | China |
|  | 50816743 | LNIJ00000000 | 410 | *blaOXA-181* | Human: blood | Norway |
|  | AMA1167 | CP024801 | 410 | *blaNDM-5 + blaOXA-181* | Human: liver | Denmark |
|  | KBN10P04869 | CP026473 | 410 | *blaNDM-5* | Human: blood | South Korea |
|  | Ecol_517 | CP018965 | 410 | *blaKPC-2* | Human: unknown | Brazil |
|  | UCI 53 | JMVS00000000 | 410 | *-* | Human: biliary drain | USA |
|  | IMT33180 | LJGI00000000 | 410 | *-* | Human: blood | Germany |
|  | 1AUH IMP194 | NEDL00000000 | 410 | *blaOXA-181* | Human: unknown | Lebanon |
|  | 105_ECOL | JWES00000000 | 38 | *-* | Human: unknown | USA |
|  | WCHE020028 | NGVE00000000 | 3052 | *blaNDM-5* | Human: unknown | China |
|  | Blood-09-0626 | JSRK00000000 | 617 | - | Human: blood | USA |
|  | 129.h | NJAE00000000 | 617 | - | Human: unknown | Nigeria |
|  | MOD1-EC5117 | NLRX00000000 | 617 | - | Human: unknown | USA |
|  | MG1655 | U00096 | 10 | - | Human: stool | USA |
| *K. pneumoniae* | 4300STDY6470411 | UFEI00000000 | 336 | *blaOXA-232* | Human: unknown | Thailand |
|  | PB21 | FLVX00000000 | 336 | *-* | Human: urine | Thailand |
|  | PB28 | FLWJ00000000 | 336 | *-* | Human: sputum | Thailand |
|  | PB298 | FLWI00000000 | 336 | *-* | Human: sputum | Thailand |
|  | 3189STDY5864816 | FXRZ00000000 | 336 | *-* | Human: urine | Pakistan |
|  | EuSCAPE RO071 | UJFC00000000 | 336 | *blaOXA-48* | Human: wound Secretion | Romania |
|  | LAU-KP1 | AYQE00000000 | 336 | *-* | Human: genitourinary | Lebanon |
|  | k1816 | FLFL00000000 | 336 | *-* | Human: blood | UK |
|  | k2169 | FLFO00000000 | 336 | *-* | Human: blood | UK |
|  | Criepir200 | WJVO00000000 | 336 | *blaOXA-48* | Human: sputum | Russia |
|  | 50675619 | LNHX00000000 | 336 | *blaNDM-7* | Human: blood | Norway |
|  | 4300STDY6636946 | UFAR00000000 | 340 | *-* | Human: unknown | Thailand |
|  | 1194 | LYZC00000000 | 340 | *blaKPC-2* | Human: catheter tip | Brazil |
|  | EuSCAPE CZ039 | UKFR00000000 | 340 | *-* | Human: wound Secretion | Czech Republic |
|  | KSB1-5D | CP024191 | 340 | *blaIMP-4* | Human: rectal swab | Australia |
|  | C1985 | SRR9722215 | 4008 | *blaKPC-2* | Human: urine | Thailand |
|  | HS11286 | CP003200 | 11 | *blaKPC-2* | Human: sputum | China |
